# Progress in understanding 2-hydroxyglutaric acidurias

**DOI:** 10.1007/s10545-012-9462-5

**Published:** 2012-03-06

**Authors:** Martijn Kranendijk, Eduard A. Struys, Gajja S. Salomons, Marjo S. Van der Knaap, Cornelis Jakobs

**Affiliations:** 1 Metabolic Unit - Department of Clinical Chemistry, VU University Medical Center, Amsterdam, The Netherlands; 2 Paediatric Neurology, VU University Medical Center, Amsterdam, The Netherlands

## Abstract

The organic acidurias d-2-hydroxyglutaric aciduria (D-2-HGA), l-2-hydroxyglutaric aciduria (L-2-HGA), and combined d,l-2-hydroxyglutaric aciduria (D,L-2-HGA) cause neurological impairment at young age. Accumulation of d-2-hydroxyglutarate (D-2-HG) and/or l-2-hydroxyglutarate (L-2-HG) in body fluids are the biochemical hallmarks of these disorders. The current review describes the knowledge gathered on 2-hydroxyglutaric acidurias (2-HGA), since the description of the first patients in 1980. We report on the clinical, genetic, enzymatic and metabolic characterization of D-2-HGA type I, D-2-HGA type II, L-2-HGA and D,L-2-HGA, whereas for D-2-HGA type I and type II novel clinical information is presented which was derived from questionnaires.

## Introduction

Gregersen et al (1977) were the first to identify enantiomeric d- and l-2-hydroxyglutaric acids (D-2-HG and L-2-HG) as normal constituents of human urine. Three years later, two novel inborn errors of metabolism were simultaneously reported in the *Journal of Inherited Metabolic Disease*. Chalmers et al (1980) identified a patient with d-2-hydroxyglutaric aciduria (D-2-HGA), while Duran et al (1980) described a case of l-2-hydroxyglutaric aciduria (L-2-HGA), landmark publications that identified the metabolic hallmarks (D- and L-2-HG) in these disorders. Muntau et al (2000) described a third biochemical variant of 2-hydroxyglutaric aciduria (2-HGA) when they reported three patients with elevated urinary D- and L-2-HG, denoted by these authors as “combined d,l-2-hydroxyglutaric aciduria” (D,L-2-HGA). Major milestones in research on these disorders came with gene discovery: *D2HGDH* encoding d-2-hydroxyglutarate dehydrogenase (D-2-HGDH)(Achouri et al 2004) and *L2HGDH* encoding l-2-hydroxyglutarate dehydrogenase (L-2-HGDH)(Rzem et al 2004; Topcu et al 2004). In many D-2-HGA, and the majority of L-2-HGA patients, genetic characterization revealed pathogenic mutations in these genes (Struys et al 2005b; Steenweg et al 2010). Nonetheless, in fully one-half of D-2-HGA patients no mutations in *D2HGDH* were detected (Kranendijk et al 2010a). Subsequently, we described gain-of-function mutations in *isocitrate dehydrogenase 2* (*IDH2*) which proved causative for the D-2-HG accumulation in previously unclassified D-2-HGA patients (Kranendijk et al 2010b). In sum, the preceding decade has provided tremendous advances in our understanding of the inborn 2-hydroxyglutaric acidurias, which will undoubtedly provide a solid foundation from which to develop novel and effective treatment strategies.

This *Review* evaluates metabolic, enzymatic, genetic and clinical progress in our understanding of the *rare inborn organic acidurias* D-2-HGA, L-2-HGA and D,L-2-HGA. Future research and therapeutic perspectives are also briefly discussed.

### Enantiomeric d,l-2-hydroxyglutaric acid and its origin

**Fig. 1:**
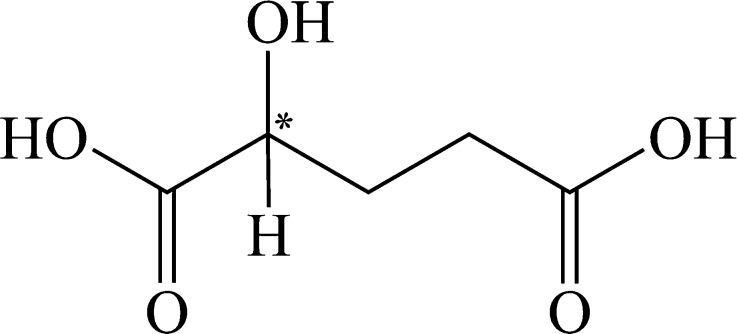
2-hydroxyglutaric acid with a chiral center at the 2nd carbon (*)

**Fig. 2:**
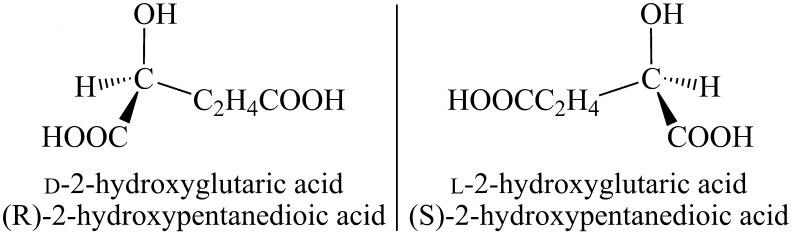
Enantiomers d- and l-2-hydroxyglutaric acid (D-2-HG and L-2-HG, systemic IUPAC names included)

The five-carbon dicarboxylic acid 2-hydroxyglutaric acid (2-HG) possesses a hydroxyl group at the second carbon (Fig. 1) which yields a chiral center. Accordingly, two three-dimensional (3D) structures exist, including D-2-HG and L-2-HG, which represent “non-superimposable” mirror images (Fig. 2). Systemic names are (R)-2-hydroxypentanedioic acid and (S)-2-hydroxypentanedioic acid, respectively, for D- and L-2-HG. Whereas enantiomers share identical chemical and physical properties (melting point, mass, solubility and pKa), their differing 3D-structures result in considerable differences in enzymatic and molecular properties.

**Fig. 3:**
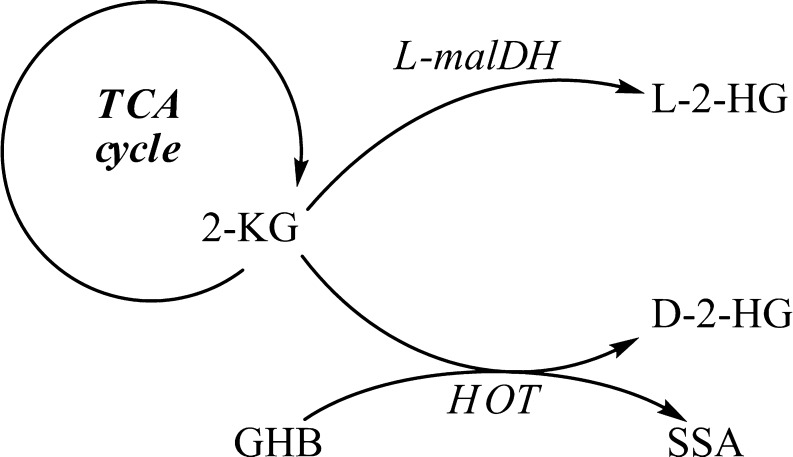
Enzymes L-malDH and HOT are responsible for production of D-2-HG and L-2-HG from 2-KG

Pilot studies employing stable isotope labeled [^13^C_6_]glucose or [^2^H_5_]glutamic acid with D-2-HGA lymphoblast cell cultures revealed that mitochondrial 2-ketoglutarate (2-KG), a tricarboxylic acid (TCA) cycle intermediate, can be metabolized to D-2-HG (Struys et al 2004b). Subsequent studies documented the existence of hydroxyacid-oxoacid transhydrogenase (HOT) activity in human liver and fibroblasts, representing the first demonstration of a human enzyme whose catalytic function was production of D-2-HG (Struys et al 2005c). HOT catalyzes the conversion of γ-hydroxybutyrate (GHB) to succinic semialdehyde (SSA) with a stoichiometric production of D-2-HG from 2-KG (Fig. 3). Similarly, pilot studies of L-2-HGA lymphoblasts incubated with [^13^C_6_]glucose and [^2^H_5_]glutamic acid further delineated that mitochondrial 2-KG is the precursor of L-2-HG (Struys et al 2007). Currently, the only enzyme known to generate L-2-HG from 2-KG in human is l-malate dehydrogenase (L-malDH) (Fig. 3), whose primary catalytic function is the interconversion of l-malate to oxaloacetate (Rzem et al 2007).

### The diagnosis of 2-hydroxyglutaric aciduria

The differential diagnosis of 2-hydroxyglutaric aciduria begins with the clinical evaluation of a patient with unexplained developmental delay and/or other neurological dysfunction of unknown etiology, raising suspicion for a metabolic disorder. Provisional diagnosis of the disorder is occasionally suggested by abnormal brain MRI findings. Urinary organic acid screening with gas chromatography-mass spectrometry (GC-MS), performed in multiple metabolic centers, can reveal increased 2-HG, but the chiral configuration remains to be determined. Although the clinical presentation often can suggest either D-2-HGA or L-2-HGA, chiral differentiation performed with GC-MS or liquid chromatography-tandem mass spectrometry (LC-MS/MS) is mandatory for the correct differential diagnosis (Gibson et al 1993a; Struys et al 2004a). Additionally, amino acid analysis in plasma and/or cerebrospinal fluid (CSF) may identify elevated lysine in L-2-HGA. Subsequently, enzymatic and genetic characterization can confirm L-2-HGA, as well as differentiate between the type I or II form of D-2-HGA, representing important information for genetic counseling and future prenatal diagnosis (Steenweg et al 2010; Kranendijk et al 2010a, b). While elevated L-2-HG levels are specific for L-2-HGA and are also detected in D,L-2-HGA, D-2-HG can be elevated in a number of other disorders in addition to D-2-HGA type I and type II, discussed in a separate section of this *Review*. Some authors have suggested that a potential false positive diagnosis of D- or L-2-HGA may occur with improperly preserved urine samples linked either to nonenzymatic conversion of 2-KG to D/L-2-HG, or via excretion of D/L-2-HG from bacterial or fungal growth in the urine specimen (Kumps et al 2002). We have not, however, experienced this occurrence in our laboratory.

## d-2-hydroxyglutaric aciduria type I and II (MIM# 600721 and MIM# 613657)

Recent studies have documented the presence of two groups, D-2-HGA types I and II, of roughly equal size and encompassing >95% of all patients. D-2-HGA type I associates with mutations in the *D2HGDH* gene encoding d-2-hydroxyglutarate dehydrogenase (D-2-HGDH) which lead to impaired enzyme function (Kranendijk et al 2010a). The type II disorder derives from specific gain-of-function mutations in the *IDH2* gene which result in accumulation of D-2-HG (Kranendijk et al 2010b). Both gene defects lead to supraphysiological accumulation of D-2-HG in urine, plasma and CSF, representing the biochemical hallmarks of the diseases.

### Clinical manifestations of D-2-HGA

D-2-HGA was recognized as a distinct neurometabolic disorder with mild and severe phenotypes in the late 1990s (Van der Knaap et al 1999a, b). The clinical phenotype encompassed epilepsy, hypotonia and psychomotor retardation as the primary features. With the genetic identification of type I (*D2HGDH* mutations, MIM# 600721) and type II (gain-of-function mutations in *IDH2*, MIM# 613657) disorders in 2010, it became clear that studies from 1999 encompassed heterogeneous groups of both subtypes of patients, as well as combined D,L-2-HGA patients, which likely led to the heterogeneous description of mild and severe phenotypes.

Herein we present novel clinical features in 14 type I and 19 type II patients based on clinical questionnaires. The age of onset for type I patients is generally within the first six years, whereas for type II the age of onset was within two years (Table 1). Cardinal clinical manifestations for both disorders include developmental delay, hypotonia and seizures, although seizures occur with higher frequency in type II patients (Table 1). All type II patients were developmentally delayed, and the delays were more severe than those observed in type I patients. In 9 of 19 type II patients cardiomyopathy (primarily dilated, but in one case hypertrophic) was observed, a feature absent from the type I phenotype. The course of the type II disease is primarily progressive, but a static disease, or even improvement have been reported in a limited number. Life expectancies for type II patients may range from several months to early adulthood (Table 1). Conversely, for type I patients the life expectancy remains undefined. One patient died in the third week of life with a diagnosis of necrotizing enterocolitis at post mortem examination, while three others are currently 5-12 years of age.

**Table 1:** Clinical observations in D-2-HGA type I and type I

	Symptoms	D-2-HGA type I	D-2-HGA type II
Number of patients		14	19
Age onset (yr)	Mean	1	¼
	Range	0-6	0-2
Signs during disease	Developmental delay	78 (11 pts.)	100 (19 pts.)
(% of type I or type II)		- 3 pts. unaffected	- 0 pts. unaffected
		- 5 pts. mild	- 2 pts. mild
		- 3 pts. moderate	- 6 pts. moderate
		- 3 pts. severe	- 11 pts. severe
	Hypotonia	57 (8 pts.)	89 (17 pts.)
	Seizures	29 (4 pts.)	79 (15 pts.)
	Cardiomyopathy	0	47 ( 9 pts.)
			- 7 pts. dilated
			- 1 pt. hypertrophic
			- 1 pt. unknown
Alive (yr)	Mean age	8 (n = 3)	8.4 (n = 10)
	Range	5, 7, 12	2.8-19
Died (yr)	Mean age	3 weeks* (n = 1)	6.5 (n = 9)
	Range	-	0.3-22
Unknown		n = 10	-

*postmortem diagnosis of necrotizing enterocolitis

The disorders appear panethnic, and consanguinity is frequent in the type I disorder while essentially absent in type II families. Clinical heterogeneity of previously published cases showed alikeness with the symptoms summarized in Table 1. Additional clinical features variably reported have included macrocephaly, dysmorphic features and cerebral visual failure. These patients were not, however, differentiated as D-2-HGA type I or type II disease. Cardiomyopathy was exclusive to type II patients in our data (Table 1), however in one reported unrelated type I patient (homozygous for c.458T>C; p.Met153Thr in *D2HGDH*) an increased cardiothoracic index and hypertrophic cardiomyopathy was documented (Haliloglu et al 2009), which is until now an isolated finding.

Neuroimaging performed in D-2-HGA patients was predominantly instituted prior to knowledge of the underlying molecular defects. The clinical features in these undifferentiated patients included enlargement of the lateral ventricles, enlarged frontal subarachnoid spaces, subdural effusions, subependymal pseudocysts, signs of delayed cerebral maturation and multifocal cerebral white-matter abnormalities (Van der Knaap et al 1999a, b). An ongoing imaging study is underway in our laboratories to more accurately define the CNS abnormalities associated with the type I and II disorders. Finally, an intriguing study of 4.5-year-old female monozygotic twins affected with type I disease (Misra et al 2005) revealed one with multiple congenital anomalies, severe developmental delay, and abnormal neuroradiological findings, while the other reached all major motor and language milestones appropriately associated with a generally uncomplicated clinical course. The latter imply that postzygotic genetic alterations and/or environmental factors influence the phenotypic outcome of the type I disease.

### *D2HGDH* mutations: the molecular basis of D-2-HGA type I

**Fig. 4:**
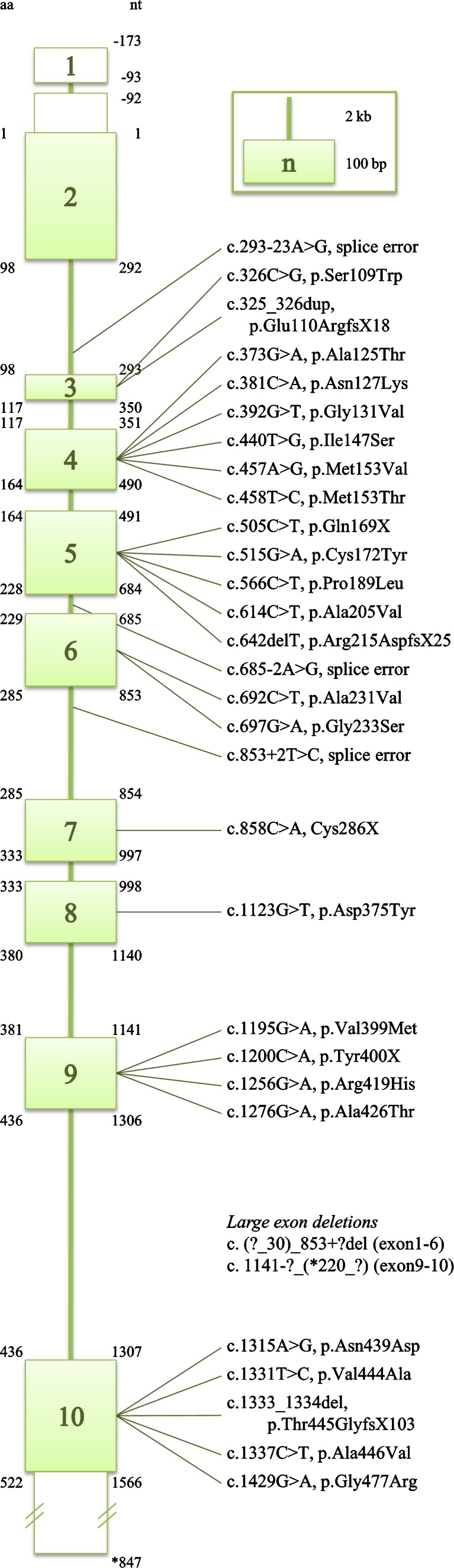
Mutations reported in *D2HGDH* (Misra et al 2005; Struys et al 2005a, b; Haliloglu et al 2009; Kranendijk et al 2010a; Pervaiz et al 2011)

Employing DEAE-sepharose chromatography combined with spectrophotometric and radioactive detection, Achouri and co-workers isolated enzymes from rat liver in order to screen for a dehydrogenase acting upon D-2-HG (Achouri et al 2004). Van Schaftingen and coworkers subsequently identified the *D2HGDH* gene (GeneBank 728294, NM_152783, MIM# 609186), which encodes a d-2-hydroxyglutarate dehydrogenase (D-2-HGDH) with considerable specificity for D-2-HG. Subsequent detection of *D2HGDH* mutations in patients with D-2-HGA confirmed the identity of this cDNA and documented the genetic lesions (Misra et al 2005; Struys et al 2005a, b; Haliloglu et al 2009; Kranendijk et al 2010a; Pervaiz et al 2011). Twenty six patients have thus far been identified, harboring 31 mutations spread across the *D2HGDH* coding sequence (Fig. 4). Eleven mutations are predicted to result in truncated enzymes, while the remaining twenty missense mutations alter amino acids conserved across species, which suggests pathogenicity. As well, limited overexpression studies of mutated alleles have confirmed their pathogenicity (Struys et al 2005b). Accordingly, patients harboring pathogenic homozygous, or compound heterozygous mutations, in the *D2HGDH* gene are denoted D-2-HGA type I (∼50% of the D-2-HGA population), and the autosomal-recessive inheritance pattern has been confirmed by genetic characterization of parental DNA samples.

### *IDH2* mutations: the molecular basis of D-2-HGA type II

Various investigators working in the field of cancer genetics identified an increased incidence of heterozygous mutations in isocitrate dehydrogenase 1 and 2 (*IDH1*, *IDH2*) in selected individuals with carcinoma. These alleles induced novel enzyme functions in IDH1 and IDH2, namely the newfound capacity to convert 2-KG to D-2-HG, and resulting in supraphysiological accumulation of D-2-HG (Parsons et al 2008; Dang et al 2009; Ward et al 2010). These data led our group to hypothesize that *IDH* mutations might represent the genetic underpinning of D-2-HGA, and we subsequently identified germline heterozygous *IDH2* mutations in a number of D-2-HGA patients (GeneBank 3418, NM_002168, MIM# 147650)(Kranendijk et al 2010b). These novel molecular events thereby represent the second D-2-HGA defect, denoted as D-2-HGA type II. We have subsequently identified 14 patients carrying the heterozygous c.419G>A, p.Arg140Gln mutation (IDH2^wt/R140Q^), in addition to one patient who was heterozygous for c.418C>G, p.Arg140Gly (IDH2^wt/R140G^). In the DNA derived from 24 patients, these gain-of-function mutations represent ∼50% of the D-2-HGA population. Moreover, *IDH2* alleles were not detected in 8 of 9 sets of parents, suggesting *de novo* occurrence and an autosomal dominant pattern of inheritance. Of interest, in one family three subsequent pregnancies were diagnosed as affected by D-2-HGA type II (genetic diagnosis in DNA isolated from amniocytes), which suggested germline mosaicism in the mother who was subsequently confirmed to harbor somatic mosaicism in blood.

Currently, 95 D-2-HGA patients have been detected (including unpublished patients identified in our laboratories), most of whom were diagnosed prior to identification of the molecular lesions (Table 2). For ∼50% of those patients, genetic characterization revealed 26 type I and 24 type II patients. In two cases no mutations were found in either gene (*D2HGDH* or *IDH2),* and thus the underlying etiology remains unknown. Several patients have previously been reported as D-2-HGA who showed (to lesser extent) increased L-2-HG as well, denoted “combined D,L-2-HGA”, which will be discussed later in detail. Another subset of D-2-HGA patients, without *D2HGDH* or *IDH2* mutations, but with skeletal dysplasia is discussed more completely later in this *Review*.

**Table 2:** Overview of number of D-2-HGA patients divided in specific groups

D-2-HGA	Remarks	References
Type I (n = 26)	1 patient affected with comorbid Sanfilippo syndrome type C	Gibson et al 1993b; Craigen et al 1994; Van der Knaap et al 1999b; Misra et al 2005; Struys et al 2005a; b; Haliloglu et al 2009; Kranendijk et al 2010a; Pervaiz et al 2011
Type II (n = 24)	15 published pts.	Geerts et al 1996; Amiel et al 1999; Van der Knaap et al 1999a; b; Clarke et al 2003; Kranendijk et al 2010b
	9 unpublished pts.*	
Undifferentiated (n = 43)	DNA unavailable for *D2HGDH* and *IDH2* sequencing	Chalmers et al 1980; Nyhan et al 1995; Sugita et al 1995; Baker et al 1997; Wagner et al 1998; Van der Knaap et al 1999a; b; Eeg-Olofsson et al 2000; Kwong et al 2002; Wang et al 2003; Mahfoud et al 2009
	18 published pts.	
	25 unpublished pts.*	
Unknown (n = 2)	D-2-HGA type I and type II were excluded: no mutations detected in *D2HGDH* or *IDH2*	Kranendijk et al 2010b
Combined	Increased D-2-HG and L-2-HG	Wagner et al 1998; Amiel et al 1999; Van der Knaap et al 1999a; b; Muntau et al 2000; Wajner et al 2002; Read et al 2005
D,L-2-HGA (n = 11)	6 published pts.	
	5 unpublished pts.*	
Skeletal dysplasia (n = 6)	3 published pts.	Talkhani et al 2000; Honey et al 2003; Bayar et al 2005
	3 unpublished pts.*	

* Unpublished patients have been diagnosed in our laboratory

### Metabolism in D-2-HGA

**Fig. 5:**
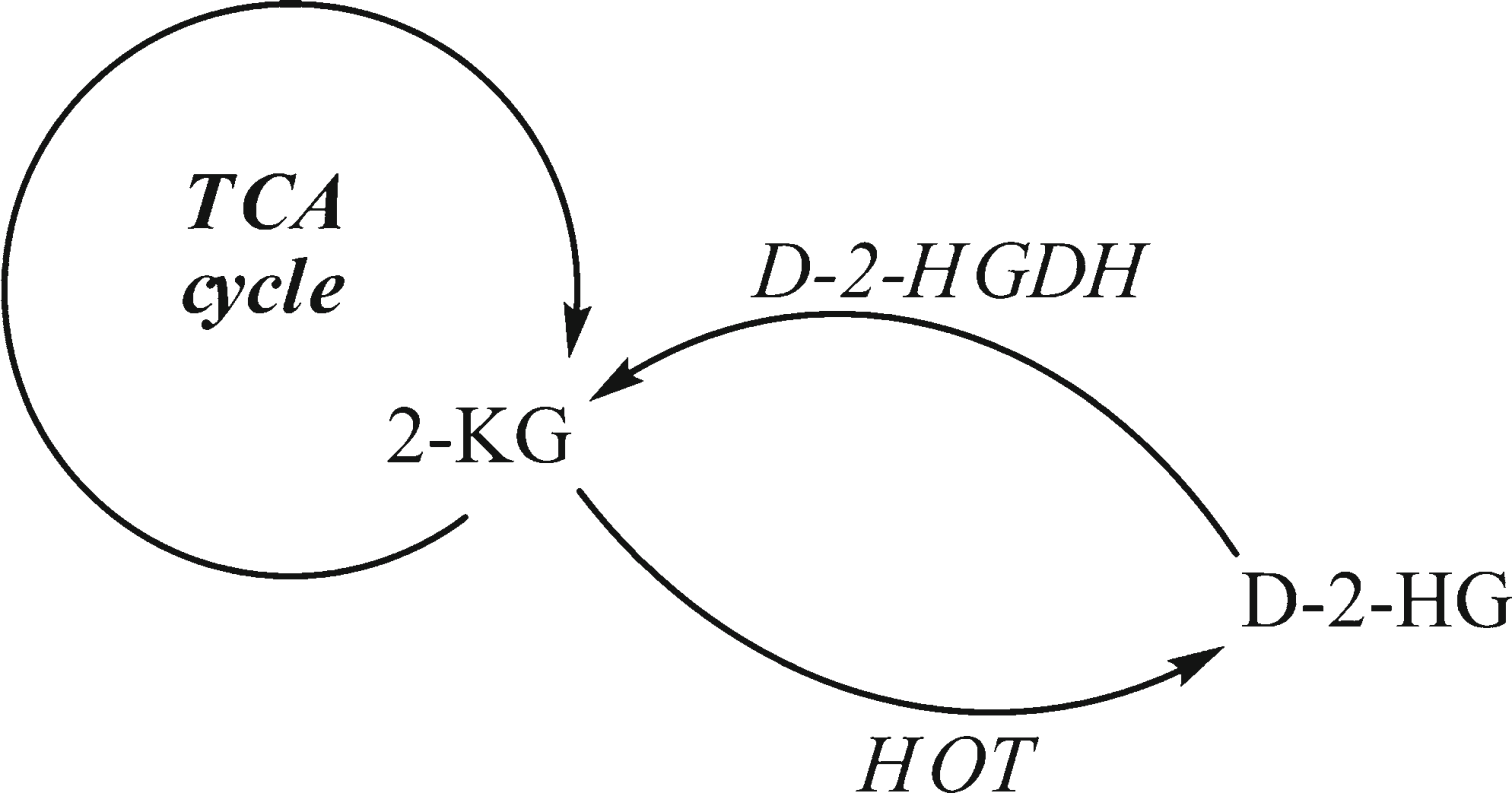
D-2-HG is formed from 2-KG via HOT. D-2-HGDH catalyzes the conversion of D-2-HG to 2-KG. D-2-HG accumulates in D-2-HGA type I patients when D-2-HGDH is impaired

Different catalytic mechanisms underlie D-2-HG accumulation in the D-2-HGA type I and II disorders. HOT (EC 1.1.99.24) converts 2-KG to D-2-HG (Fig. 5)(Struys et al 2005c), and currently there is no known physiological function for D-2-HG in human metabolism. To maintain carbon balance and avoid intoxication, D-2-HG is interconverted to 2-KG via D-2-HGDH (EC 1.1.99.-). Our laboratory demonstrated impaired D-2-HGDH activity in lymphoblasts and fibroblasts derived from type I patients, whereas this activity was normal in type II cells (Table 3) (Wickenhagen et al 2009; Kranendijk et al 2010a). Thus, D-2-HG accumulation in the type I disease is directly correlated with deficient D-2-HGDH activity.

**Table 3:** Enzyme activities of D-2-HGDH and IDH2^wt/R140Q^ in D-2-HGA cell lines

	D-2-HGDH fib. (pmol h^-1^ mg prot.^-1^)	D-2-HGDH lyb. (pmol h^-1^ mg prot.^-1^)	IDH2^wt/R140Q^ lyb. (pmol h^-1^ mg prot.^-1^)
	mean (n); range	mean (n); range	mean (n); range
Controls	456 (5); 247-665	1409 (5); 273-2545	1800 (5); 1400-2100
D-2-HGA type I	22 (5); 0-41	12 (2); 2, 21	1900 (2); 1900, 1900
D-2-HGA type II	338 (14); 204-634	1062 (4); 570-1503	14400 (5); 12000-18800

fib. = fibroblasts; lyb. = lymphoblasts (Wickenhagen et al 2009; Kranendijk et al 2010a, 2011)

**Fig. 6:**
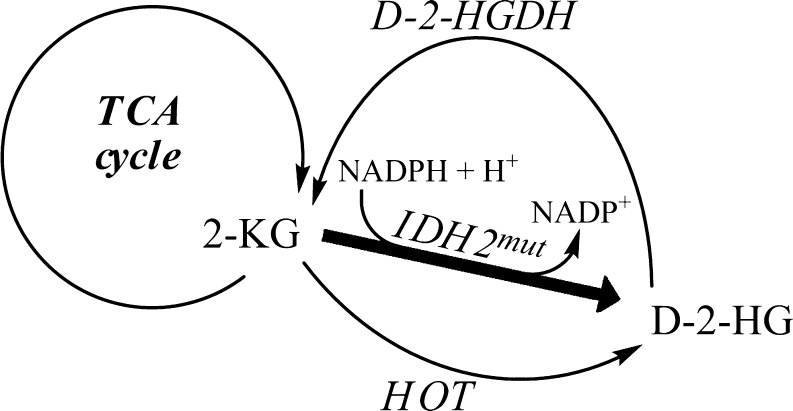
Novel IDH2-mutant gain-of-function produces D-2-HG from 2-KG in addition to production of D-2-HG via HOT. It is hypothesized that D-2-HGDH cannot fully metabolize all of the generated D-2-HG, resulting in D-2-HG accumulation in D-2-HGA type II

In the type II disorder, heterozygous mutations detected in isocitrate dehydrogenase 2 (IDH2, EC 1.1.1.42) at residue 140 replaces arginine by glutamine or glycine (Kranendijk et al 2010b). While wild type IDH2 reversibly catalyzes the conversion of isocitrate to 2-KG using the NADP(H) couple, IDH2^wt/R140Q^-mutant acquires the enzymatic capacity to convert 2-KG to D-2-HG using NADPH as a hydride donor (Fig. 6). Along these lines, an eight-fold increase of IDH2^wt/R140Q^ reaction velocity was detected in lymphoblasts obtained from type II patients in comparison to control and type I cells (Table 3) (Kranendijk et al 2011). We have speculated that the capacity of active D-2-HGDH is insufficient to metabolize the excess D-2-HG formed by gain-of-function IDH2^wt/R140Q^.

Different mechanisms leading to the production of D-2-HG in the type I and II disorders appear to be reflected in the absolute concentrations of this metabolite in physiological fluids and intracellularly in cultured lymphoblasts (Table 4) (Kranendijk et al 2010a, b; Kranendijk et al 2011). D-2-HG levels are 2 to 8-fold higher in type II as opposed to type I patients, although the absolute level of D-2-HG is highly increased in all fluids, or cells, that were examined. Metabolite concentrations in plasma exceed those in CSF for both groups. Moreover, extensive metabolic screening (e.g., organic acids, amino acids, acylcarnitines, GABA) has been performed in multiple physiological fluid samples derived from patients, yet the only consistent biochemical marker remains D-2-HG in both subtypes, with normal levels of L-2-HG in all instances.

**Table 4:** D-2-HG concentration in body fluids and cultured lymphoblasts of D-2-HGA patients

	Urine (mmol/mol creat.)	Plasma (μmol/L)	CSF (μmol/L)	Lymphoblasts (nmol/mg prot.)
	mean (n); range	mean (n); range	mean (n); range	mean (n); range
Controls	6 (18); 2.8-17	0.7 (10); 0.3-0.9	0.1 (10); 0.07-0.3	0.11 (5); 0.06-0.13
D-2-HGA type I	969 (20); 103-2414	68 (7); 26-123	13 (3); 6-18	1.8 (2); 1.7-1.9
D-2-HGA type II	2486 (19); 448-11305	366 (9); 99-757	79 (4); 30-172	15.1 (5); 1.9-28.5

(Gibson et al 1993b; Kranendijk et al 2010a,b, 2011)

### Pathophysiology of D-2-HGA

Although genetically distinct, D-2-HGA types I and II share accumulation of D-2-HG in physiological fluids, as well as common clinical features, but without cardiomyopathy in type I. These observations suggest that the metabolite D-2-HG contributes to the pathophysiology associated with the clinical features of developmental delay, hypotonia and seizures observed in both groups. Overproduction of D-2-HG likely begins in the mitochondria, since D-2-HGDH and IDH2 are mitochondrial enzymes. Intracellular/mitochondrial D-2-HG concentrations are unknown, but plasma concentrations are often used as a surrogate measure. Plasma D-2-HG concentrations are 30-840 fold increased in patients (26-757 μM)(Table 4). The mean plasma levels are ∼5 times higher in type II than in type I patients with little overlap of the ranges. Since the frequency and severity of developmental delay, hypotonia and seizures observed in type II patients are slightly higher than those observed in the type I disorder (Table 1), there appears to be a correlation between increasing D-2-HG concentrations and disease severity.

In vitro studies have revealed both cyto- and neurotoxic effects of high levels of D-2-HG. Along these lines, exposure of tissues from rat and chick to increasing D-2-HG concentrations downregulated the creatine kinase, complex IV and complex V enzymes (da Silva et al 2002; Kölker et al 2002; da Silva et al 2003a, b, 2004). Additionally, high levels of D-2-HG induced oxidative stress and markedly impaired mitochondrial energy metabolism in several model systems in vitro (Kölker et al 2002; Latini et al 2003b, 2005). Neurotoxic effects of increased D-2-HG was revealed by increased synaptosomal glutamate uptake without alteration in other synaptosomal parameters studied (Junqueira et al 2004), as well as by NMDA receptor activation in primary neuronal cultures (Kölker et al 2002).

The IDH-mutated gain-of-function allele received considerable attention in the broad areas of cancer research, since it was first identified in brain tumors (Parsons et al 2008). Of interest, neoplastic disorders have not been reported in either D-2-HGA type I or II patients, which would argue against the hypothesis proposed by others that D-2-HG is an onco-metabolite (Dang et al 2009; Ward et al 2010). On the other hand, Zhao et al (2009) demonstrated that the hypoxia-inducible factor subunit-1α (HIF-1α), a transcription factor that facilitates tumor growth in the presence of low oxygen, was higher in human gliomas harboring an *IDH1* mutation than tumors lacking a mutation. Xu et al (2011) reported that D-2-HG acts as a competitive inhibitor of multiple 2-KG-dependent dioxygenases which leads to a genome-wide alteration in histone and DNA methylation patterns. These authors suggested that the latter may hypothetically contribute to tumorigenesis through alterations of epigenetic control and changes in stem cell differentiation. The preceding observations, focused on epigenetic effects and outcomes, could be in line with our findings in monozygotic twins affected with D-2-HGA type I (Misra et al 2005). Random affects of gene methylation combined with environmental influences during pregnancy and/or after birth might influence phenotypic outcomes, although the twins are genetically identical.

### Therapeutic perspectives

There is currently no effective therapeutic intervention for D-2-HGA. Addition of FAD to purified D-2-HGDH enzymes did not result in increased activity (Achouri et al 2004). A therapeutic trial with riboflavin has not been attempted, but might be beneficial since the DNA sequence of *D2HGDH* belongs to a family of enzymes that use FAD as a cofactor. In the type II disease, inhibition of IDH2-mutants with oxaloacetate led to decreased D-2-HG production in patient lymphoblast lysates (Kranendijk et al 2011). Inhibition with highly IDH2-mutant specific substrates/drugs may represent a viable therapeutic strategy in patients.

## l-2-hydroxyglutaric aciduria (MIM# 236792)

l-2-hydroxyglutaric aciduria (L-2-HGA) is an autosomal recessive encephalopathy. It is a “disorder of metabolite repair” (Van Schaftingen et al 2008), but the pathophysiology of the white matter abnormalities is poorly understood.

### Clinical manifestations of L-2-HGA

Several clinical reports of L-2-HGA have appeared since the index case in 1980 (Barth et al 1993; Barbot et al 1997; Achouri et al 2004; Topcu et al 2005; Steenweg et al 2009, 2010). The phenotype is homogeneous (Table 5). Steenweg and co-workers described the largest cohort of 106 patients. An insidious onset of disease starting in childhood, developmental delay, epilepsy and cerebellar ataxia were the cardinal clinical signs (Steenweg et al 2010). Accordingly, the course of the disease is slowly progressive. Therefore, mild L-2-HGA patients often remain undiagnosed until adolescence or even adulthood. Virtually all patients display delayed mental and motor development, and about two-third of them epilepsy and cerebellar dysfunction. In about half of patients, macrocephaly and extrapyramidal symptoms including tremor and dystonia are observed. Hypotonia was most prevalent in the earlier stages and spasticity in the latter stages of disease in their cohort. Neurological decompensation (e.g., loss of milestones, such as unassisted walking and the development of speech deficits) was also present in a quarter of the patients.

**Table 5:** Clinical observations in L-2-HGA reported in the literature

	Barth et al 1992, 1993	Barbot et al 1997	Topcu et al 2005	Steenweg et al 2009, 2010
Number of patients- clinical description	12	6	29	106
Age onset	After infancy	½-2 yr (100%)	1-10 yr (67%)	0-7 yr (97%)
			11-18 yr (24%)	
			19-30 yr (7%)	
Insidious onset	+	+		+
Signs at diagnosis				
developmental delay	+ (33%)	+ (83%)	+	+ (52%)
epilepsy	+ (1 pt.)	+ (1 pt.)		+ (42%)
cerebellar ataxia	+ (1 pt.)		+	+ (20%)
Signs during disease course				
developmental delay	+ (100%)	+ (100%)	+ (79%)	+ (93%)
cerebellar ataxia	+ (92%)	+ (100%)	+ (66%)	+ (82%)
epilepsy	+ (50%)	+ (67%)	+ (41%)	+ (72%)
macrocephaly			+ (52%)	+ (48%)
extrapyramidal symptoms	+ (33%)			+ (38%)
Progression	Chronic, slowly	Slowly	Static	Slowly
Number of patients - brain MRI performed	10	6	24	56
Highly characteristic MRI abnormalities*	+	+	+	+

*Combination of predominantly subcortical cerebral white matter abnormalities and abnormalities of the dentate nucleus, globus pallidus, putamen, and caudate nucleus

Steenweg et al (2009) systematically evaluated the brain MRIs of 56 patients, and found a highly characteristic pattern of MRI abnormalities in L-2-HGA including abnormalities of the subcortical cerebral white matter dentate nucleus, globus pallidus, putamen, and caudate nucleus. These results correlated well with previous case reports of L-2-HGA patients (Barth et al 1993; Barbot et al 1997; Topcu et al 2005). As the disease progresses, abnormalities of white matter and basal ganglia signal intensities became more diffuse, followed by cerebral white matter atrophy. Barbot et al (1997) found a strong correlation between the severity of clinical manifestations and the extent of the MRI lesions in a cohort of six patients, but this correlation was not noted by Steenweg et al (2009).

Acute metabolic derangement has not been reported in L-2-HGA, although rapid deterioration has been noted in nine cases, in relation to brain tumors (Moroni et al 2004; Haliloglu et al 2008; Aghili et al 2009). Additionally, Vilarinho et al (2005) reported two children and one adult with brain astrocytomas in their series of 21 L-2-HGA patients of Portuguese descent, while Steenweg et al (2009) mentioned only a single case affected with a tumor in the cerebral cortex in their cohort of 56. The preceding 13 cases of patients with brain tumors suggest an association of L-2-HGA with CNS tumors, which has led to speculation that L-2-HG may predispose to oncogenesis. Non-CNS tumors have been documented in L-2-HGA as well, including a bone tumor involving the right frontal region of the calvaria in one patient (Larnaout et al 2007) and nephroblastoma (Wilms tumor) in a second patient (Rogers et al 2010). On the other hand, a subsequent study in 21 Wilms tumor tissues did not reveal increased L-2-HG levels, which therefore did not directly relate L-2-HG with Wilms tumor formation (Rakheja et al 2011). The possible association of the metabolite L-2-HG and tumorigenesis requires further investigation.

### L2HGDH mutations: the molecular basis of L-2-HGA

Two research groups independently identified the gene associated with L-2-HGA. Rzem et al (2004) purified a dehydrogenase from rat liver that catalyzed the conversion of L-2-HG to 2-KG. Various assay methodologies identified several properties of the proposed l-2-hydroxyglutarate dehydrogenase (L-2-HGDH) which facilitated database searches and an eventual identification of the *L2HGDH* gene (GeneBank 79944, NM_024884, MIM# 609584). Subsequently, homozygosity mapping in three unrelated consanguineous families with L-2-HGA confirmed the association between the *L2HGDH* gene and l-2-hydroxyglutaric aciduria. An additional homozygosity mapping study identified *C14orf160*, a gene carrying mutations in 21 confirmed L-2-HGA patients from 15 Turkish families (Topcu et al 2004).

Numerous mutations in *L2HGDH* have now been reported worldwide, summarized in a *Mutation Update* (Steenweg et al 2010) and registered in the *Leiden Open Variation Database* (www.LOVD.nl/L2HGDH 2011). Currently, 86 unique variants have been described in 164 individuals who are homozygous or compound heterozygous (157 index, seven siblings) for these alleles. The majority of variants are missense mutations that alter invariably conserved amino acids.

### Metabolism in L-2-HGA

**Fig. 7:**
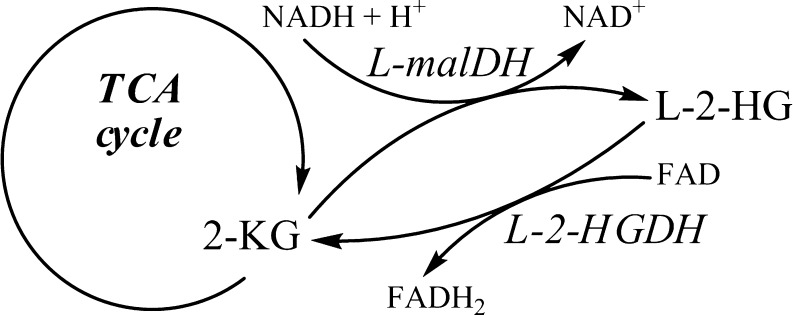
L-2-HG is formed via the non-specific interaction of L-malDH with 2-KG using NADH. The “enzyme of metabolite repair” L-2-HGDH catalyzes the interconversion of L-2-HG to 2-KG using FAD as cofactor. L-2-HG accumulates in L-2-HGA patients when L-2-HGDH is impaired

L-2-HG is formed from 2-KG in a non-specific reaction catalyzed by l-malate dehydrogenase (L-malDH, EC 1.1.1.37), which employs NADH as the hydride donor (Fig. 7) (Rzem et al 2007). This reaction is hypothesized to be an “unwanted” side reaction of L-malDH, which normally catalyzes the conversion of l-malate to oxaloacetate in the TCA cycle. Accordingly, to prevent loss of carbon moieties in the TCA cycle, and to protect against potential toxic effects, L-2-HG is reconverted to 2-KG via L-2-HGDH (EC 1.1.99.2), with FAD as cofactor. The role of L-2-HGDH, therefore, appears to be that of a “repair” mechanism for this unwanted side reaction of L-malDH, since L-2-HG has no known physiological function in human. L-2-HGA can thus be considered a “disorder of metabolite repair” (Van Schaftingen et al 2008).

The biochemical hallmark of L-2-HGA is the accumulation of urinary L-2-HG, which is 10 to 300 times increased in comparison to controls (Table 6), but correlation of excretion levels with disease severity have not been demonstrated (Barbot et al 1997; Steenweg et al 2010). The relationship between molecular variants and biochemical abnormalities (increased L-2-HG) in L-2-HGA was confirmed with a specific L-2-HGDH enzyme assay employing stable isotope labeled l-[3,3,4,4-^2^H_4_]2-hydroxyglutaric acid and LC-MS/MS (Kranendijk et al 2009). Impaired L-2-HGDH enzyme activity was documented in fibroblasts, lymphoblasts and lymphocytes derived from 15/15 L-2-HGA patients, and in only one residual activity was detected (20% of control). Of interest, this patient was compound heterozygous for two missense mutations, which is considered a relatively mild genotype. On the other hand, some interesting associations have been observed between *L2HGDH* variants and urinary L-2-HG concentrations. Patients harboring two missense mutations excreted 25-50% less L-2-HG than those with two presumed null mutations (Table 6), suggesting residual enzyme activity in the former group (Steenweg et al 2010). Transfection studies in human embryonic kidney cells overexpressing human *L2HGDH* have confirmed the absence of L-2-HGDH enzyme activity when pathogenic mutations were incorporated (Rzem et al 2006). L-2-HG levels in plasma and CSF are elevated as well, with a CSF/plasma ratio >1 (Table 7). This is contrarily to the findings in D-2-HGA where D-2-HG concentrations in CSF are lower than in plasma (ratio CSF/plasma <1, Table 4).

**Table 6:** Urinary L-2-HG concentrations in reported patients and controls

L-2-HGA patients (n)	Urinary L-2-HG (mmol/mol creat.)	References
*Controls (18)*	*1.3- 19*	Gibson et al 1993a
L-2-HGA (9)	332-2742	Gibson et al 1993a
L-2-HGA (12)	226-4299	Barth et al 1993
L-2-HGA (7)	630-1420	Barbot et al 1997
L-2-HGA (29)	1000-5520	Topcu et al 2005
L-2-HGA (15)	671-3392	Kranendijk et al 2009
L-2-HGA (106)	350-3357	Steenweg et al 2010
L-2-HGA (mutations *L2HGDH*)		Steenweg et al 2010
c.905C>T (6)	1090 p = 0.012^*#*^	
c.530_533delinsATT (9)	2147	
L-2-HGA (mutations *L2HGDH*)		Steenweg et al 2010
missense mutations (28)	1431 p = 0.012^*#*^	
presumed null mutations (28)	1916	

^#^ Significant differences were found between L-2-HGA patient groups carrying a “mild” missense mutation compared to a “severe” presumed null mutation, showing 25-50% lower urinary L-2-HG levels in the former group

**Table 7:** L-2-HG and lysine concentrations in urine, plasma and CSF of reported patients

Mean (n); range	Urine (mmol/mol creatinine)		Plasma (μmol/L)		CSF (μmol/L)	
	Controls	L-2-HGA	Controls	L-2-HGA	Controls	L-2-HGA
L-2-HG						
Gibson et al 1993b	6 (18); 1.3-19	1283 (9); 332-2742	0.6 (10); 0.5-1.0	47 ( 8); 27-62	0.7 (10); 0.3-2.3	62 (6); 34-100
Barth et al 1993	<52	1810 (12); 226-4299	n.d.	31 (10); 7-84	n.d.	122 (6); 23-474
Barbot et al 1997	<15	1000 (6); 650-1420	*-*	#	*-*	#
mean (n); range		1364 (27); 226-4299		39 (18); 7-84		92 (12); 23-474
Lysine						
Gibson et al 1993b	*-*	-	*-*	-	*-*	-
Barth et al 1993	*7-45*	27 (4); 11-42	120-230	279 (8); 70-380	10-25	79 (6); 66-89
Barbot et al 1997	*7-58*	89 (6); 36-168	40-163	285 (6); 185-396	14-25	77 (2); 60, 95

n.d. = not detected; ^#^increased values were detected in plasma and CSF in one patient, as well as an increased CSF/plasma ratio

In addition to L-2-HGA, lysine is also moderately elevated in CSF and plasma derived from patients (Table 7). Increased urinary levels of lysine were identified in 4 out of 6 patients (Barbot et al 1997), but Barth et al (1993) failed to demonstrate this abnormality in the cohort those investigators studied. Direct correlations between L-2-HG and lysine levels in any physiological fluid have not been documented. Kamoun et al (2002) noted that hyperlysinemia (MIM #238700) is observed secondarily in disorders in which 2-KG is decreased. Since the ε-amino group of lysine is transaminated to 2-KG through the intermediate saccharopine to form 2-aminoadipic semialdehyde and glutamic acid, representing a two step mitochondrial process involving alpha-aminoadipic semialdehyde synthase (AASS), these authors have suggested that lysine accumulation in L-2-HGA reflects low mitochondrial 2-KG availability.

### Pathophysiology of L-2-HGA

Exposure of rat brain tissues to increased L-2-HG significantly inhibited creatine kinase activity in rat cerebellum homogenates (da Silva et al 2003c), induced oxidative stress (Latini et al 2003a) and increased glutamate uptake in synaptosomes and synaptic vesicles (Junqueira et al 2003), indicating a potential neurodegenerative effect of L-2-HG. Similar results were previously observed with high levels of D-2-HG, so this outcome is not specific for L-2-HG. Conversely, the characteristic pattern of brain MRI abnormalities in L-2-HGA supports the hypothesis that a specific pathophysiological mechanism exists in L-2-HGA, probably L-2-HG concentration-dependent, but this remains to be conclusively demonstrated. The role of lysine accumulation in the disease pathology remains uncertain, since hyperlysinemia is observed in impaired AASS enzyme activity, a rare metabolic disorder considered to be a “nondisease”, since patients generally achieve normal intellectual performance (Dancis et al 1983; Saudubray and Rabier 2007).

### Therapeutic approaches

Specific therapeutic approaches in L-2-HGA have not been reported, although two anecdotal reports have provided provocative results and potential avenues for exploration. L-2-HGA was documented in a 40-year-old female with mild limb spasticity, marked dystonia of the neck and arms, mild intellectual delay, and a homozygous mutation in *L2HGDH* (Samuraki et al 2008). Brain MRI revealed diffuse atrophy and leukoencephalopathy involving mainly subcortical white matter. Intervention with FAD (30 mg/day) and levocarnitine chloride (900 mg/day) was instituted, resulting in gradual improvement in tremor and dystonia within weeks. After six months of intervention, gait was normal and urinary L-2-HG had decreased by 50%, and this improvement was maintained for more than 4 years following initiation of treatment.

The second patient was a 16-year-old boy with L-2-HGA who presented developmental delay since infancy, but alterations in *L2HGDH* were not reported. He was unable to walk without support until 3 years of age (Yilmaz 2009), associated with impaired language skills and difficulties with hand movements. Cognitive function became increasingly impaired with age. Intervention with riboflavin (vitamin B2, precursor of FAD; 100 mg/day) partially improved cognitive and motor performance within days, and the urinary L-2-HG level decreased by 75% within three months of treatment. Cessation of riboflavin treatment resulted in significant decompensation (including clinical symptoms and urinary L-2-HG excretion). Reinstitution of riboflavin intervention returned the patient to his previously improved clinical picture within days. His brain MRI showed leukodystrophic changes prior to therapy, which remained unchanged after two years of riboflavin intervention.

In contrast to these two patients, a two month therapeutical trial with riboflavin (200 mg/day) was unsuccessful in a 9-year-old female with L-2-HGA (Jequier et al 2008). She developed a progressive action tremor, light gait ataxia, dysarthria and moderate mental retardation associated with a homozygous splice site mutation in *L2HGDH*. L-2-HG levels in physiological fluids remained unchanged, suggesting that riboflavin was unable to augment a complete absence of residual enzyme activity associated with the splicing variant.

The fact that L-2-HGDH employs FAD as co-substrate (Fig. 7) and that its enzyme increases with increasing FAD concentrations (Rzem et al 2006), suggest that FAD acts in a chaperone function to restore L-2-HGDH enzyme activity and thereby reduces L-2-HGA excretion. Nevertheless, it appears that this approach (FAD supplementation) is only effective in “mild” missense mutations in *L2HGDH* as found in the first patient, whereas truncated enzymes (presumed null mutations) are not responsive (Table 6). One patient, having compound heterozygous missense mutations in *L2HGDH*, was found to have modest residual L-2-HGDH enzyme activity (Kranendijk et al 2009), but a therapeutic trial with riboflavin has not been undertaken. Unfortunately, riboflavin intervention is not expected to improve the leukoencephalopathy observed in patients.

## Combined d,l-2-hydroxyglutaric aciduria

Currently, six cases of d,l-2-hydroxyglutaric aciduria (D,L-2-HGA) have been described, of which four were previously classified as D-2-HGA affected with a severe phenotype (Wagner et al 1998; Amiel et al 1999; Van der Knaap et al 1999a, b; Muntau et al 2000; Wajner et al 2002; Read et al 2005). D,L-2-HGA is biochemically characterized by moderately increased D-2-HG and L-2-HG in urine, mild increases of both metabolites in plasma, and a very slight elevation of D-2-HG in CSF and normal levels of L-2-HG (Table 8). D-2-HG concentration exceeds L-2-HG in all fluids, while lactate and TCA cycle metabolites (succinate, fumarate and malate) were variably increased in urine, and 2-ketoglutarate was consistently increased in four patients (A-D).

**Table 8:** Biochemical findings in combined D,L-2-HGA patients reported in the literature

Patient	Urine (mmol/mol creatinine)		Plasma (μmol/L)		CSF (μmol/L)	
	D-2-HG	L-2-HG	D-2-HG	L-2-HG	D-2-HG	L-2-HG
*Controls*	*2.8-17*	*1.3-19*	*0.3-0.9*	*0.5-1.0*	*0.07-0.3*	*0.3-2.3*
A	315-1185	162-332	2.5	2.3-3.7	2.5	1.2
B	520	142	2.22	1.07	0.49	normal
C*	228	145	--	--	--	--
D*	17.9-1072	25.2-430	2.48	2.22	0.42	normal
E	632, 685, 786	32, 76, 83	--	--	--	--
F	162, 306	127, 152	1.8, 4.6	1.91, 1.7	--	--
mean (n); range	496 (6); 228-750	161 (6); 64-247	2.6 (4); 2.2-3.2	2.0 (4); 1.1-3.0	1.1 (3); 0.42-2.5	normal (3)

* Siblings; References: *Controls*-(Van der Knaap et al 1999a); A-(Wagner et al 1998) case2, (Van der Knaap et al 1999b) pat.8, (Muntau et al 2000) pat.3; B-(Amiel et al 1999) case2, (Van der Knaap et al 1999a) pat.4; C-(Muntau et al 2000) pat.1, (Van der Knaap et al 1999a) pat.1; D-(Muntau et al 2000) pat.2, (Van der Knaap et al 1999a) pat.2; E-(Wajner et al 2002); F-(Read et al 2005)

Five patients (A-D, F) manifested a consistent clinical picture encompassing severe neonatal epileptic encephalopathy, often accompanied by respiratory insufficiency requiring artificial ventilation (Table 9). Four probands died within the first year, and the fifth at 3.5 years. The brain MRI of all revealed enlarged ventricles, subependymal pseudocysts and delayed gyration and myelination. Patient E additionally manifested hypotonia, developmental delay, seizures, cardiomyopathy and respiratory distress, with a brain MRI suggestive of mitochondrial disease (Wajner et al 2002). The patient expired at 10 months of age in cardiogenic shock. The clinical features of patient E show similarities with D-2-HGA type II, although the increased L-2-HG and brain MRI abnormalities deviate with those of D-2-HGA type II. Therefore we speculate that the etiology in this patient is different from that of the other patients.

**Table 9:** Clinical observations in D,L-2-HGA patients reported in the literature

	Patient A	Patient B	Patient C*	Patient D*	Patient E	Patient F
Gender	Female	Female	Male	Female	Male	Female
Age at death	8 months	<11 months	3.5 years	2.5 months	10 months	1 month
Consanguinity	-	-	+	+	-	-
Epileptic encephalopathy	+	+	+	+	+	+
Developmental delay	Severe	Severe	Severe	Severe	Severe	Severe
Respiratory insufficiency	+		+	+	+	
Other signs		Facial dysmorphism			Hypotonia, cardiomyopathy	
Brain MRI abnormalities	+	+	+	Not performed	Suggestive of mitochondrial disease^#^	+
Enlarged ventricles	+	+	+			+
Subependymal pseudocysts	+	+	+			+
Delayed gyration and myelination	+		+		+	+
References	Wagner et al 1998	Amiel et al 1999	Muntau et al 2000	Muntau et al 2000	Wajner et al 2002	Read et al 2005
	Muntau et al 2000					

* Siblings; ^#^ Brain MRI revealed bilateral lesions in the substantia nigra, the periaqueductal area, the medial part of the thalamus, the hypothalamus, the caudate nucleus, putamen and globus pallidus

We speculate that increased 2-KG in patients with D,L-2-HGA lead to the formation of D-2-HG and L-2-HG via HOT and L-malDH activities (Fig. 3). Nonetheless, the D,L-2-HGA disorder appears distinct from D-2-HGA and L-2-HGA, with respect to the much lower level of both isomers in physiological fluids, as well as the presence of increased lactate and TCA cycle intermediates (2-KG, succinate, fumarate and malate), suggestive of mitochondrial dysfunction. No mutations were detected in either *D2HGDH*, *L2HGDH*, or *IDH* (unpublished data) from patients B and F. We speculate that the mode of inheritance in D,L-2-HGA is autosomal recessive since patients C and D were from a consanguineous union. Currently, treatment options are unavailable for combined D,L-2-HGA.

## Other disorders with accumulation of 2-HG

### Neoplastic disorders with *IDH* mutations

Mutations in *isocitrate dehydrogenase 1* and *2* (*IDH1*, *IDH2*), confer on these enzymes a new function, namely the capacity to convert 2-KG to D-2-HG (Parsons et al 2008; Dang et al 2009; Ward et al 2010). This mechanism is identical to that observed in D-2-HGA type II. Consequently, elevated D-2-HG has been observed in samples of malignant gliomas and tumor tissues (Dang et al 2009), as well as in cells and serum obtained from acute myeloid leukemia patients (Ward et al 2010; Gross et al 2010; Sellner et al 2010). All of these pathological conditions showed mutations in either *IDH1* or *IDH2*. Many investigators suggest that D-2-HG is an onco-metabolite, but this has not been proven.

### D-2-HGA in skeletal dysplasia (MIM# 271550)

Three patients with skeletal dysplasia manifested increased D-2-HG excretion, a feature not previously reported in either D-2-HGA type I or II. Talkhani et al (2000) reported a patient with spondyloenchondrodysplasia and elevated D-2-HG in urine and plasma, and normal L-2-HG concentration. The proband was diagnosed at 1 year with a crippling form of skeletal dysplasia, and global developmental delay that improved in her second year of life. The second patient was a male infant diagnosed at 6 months with skeletal dysplasia, and mild motor and mental delay (Honey et al 2003). Urinalysis revealed increased D-2-HG. At 11 months the cardinal clinical feature was spondyloenchondrodysplasia associated with hypotonia, delayed fine motor function, and severely delayed speech development. Brain MRI revealed moderate dilatation of the lateral ventricles, a large cavum septi pellucidi and cavum vergae, with normal white matter maturation. The third proband was a 17-month-old boy with waddling gait and swollen joints, whose motor and mental development (other than delayed walking) was normal at 3 years of age (Bayar et al 2005). A bone survey revealed severe metaphyseal widening, splaying, cupping and fragmentation. D-2-HG was elevated in the urine on two occasions. The clinical and metabolic similarities led Bayar and colleagues to label it as “metaphyseal enchondrodysplasia with d-2-hydroxyglutaric aciduria”, since this cohort of patients distinctly differ radiographically and biochemically from other cases of spondyloenchondrodysplasia.

Very recently, Vissers et al (2011) detected in four patients affected with metaphyseal chondromatosis and d-2-hydroxyglutaric aciduria (MC-HGA), including the patient reported by Bayar et al, somatic mutations of IDH1 (p.Arg132His, p.Arg132Ser) using exome sequencing of blood DNA. These somatic mutations in IDH1 may explain all features of MC-HGA, including the sporadic occurrence, metaphyseal disorganization and chondromatosis, urinary excretion of D-2-HG, and reduced cerebral myelinization.

### Multiple Acyl-CoA dehydrogenase deficiency (MADD, MIM# 231680, glutaric aciduria type II)

MADD is an autosomal recessively-inherited disorder of fatty acid, amino acid, and choline metabolism (Olsen et al 2007; Liang et al 2009). MADD can result from defects in two flavoproteins: electron transfer flavoprotein (ETF) or ETF:ubiquinone oxidoreductase (ETF:QO). ETF is an electron acceptor for several dehydrogenases, subsequently transferring these to ETF-QO which then moves these electrons to ubiquinone further along in the respiratory chain. In MADD, glutaric aciduria is the biochemical hallmark, the elevated D-2-HG is often observed in urine. The pathophysiology is thought to involve impaired D-2-HGDH activity, since it cannot transfer its electrons to defective ETF or ETF:QO.

### Succinic semialdehyde dehydrogenase deficiency (SSADH, MIM# 271980)

SSADH deficiency is an autosomal recessively inherited disorder caused by mutations in the *SSADH* gene (Jakobs et al 1993; Akaboshi et al 2003). SSADH catalyzes the conversion of succinic semialdehyde (SSA) to succinic acid (SA). SSA accumulates and is converted to 4-hydroxybutyrate (GHB) via SSA-reductase. GHB represents the biochemical hallmark of this disease. The proposed pathophysiology is that elevated intracellular GHB drives the HOT reaction to form SSA and increased D-2-HG in stoichiometric conversion from 2-KG (Fig. 3) (Struys et al 2006).

### Miscellaneous disorders related with 2-HG

Other disorders show increased levels of 2-HG (undifferentiated for D-2-HG or L-2-HG, or combined accumulation of both), often related to a primary accumulation of 2-KG. For example, DOOR-syndrome (an autosomal recessive malformation syndrome) variably features elevated urinary 2-KG and 2-HG (James et al 2007). Also dihydrolipoyl dehydrogenase (E3) deficiency manifests increased levels of many organic acids, including 2-KG and 2-HG (Kuhara et al 1983).

## Final remarks

**Fig. 8:**
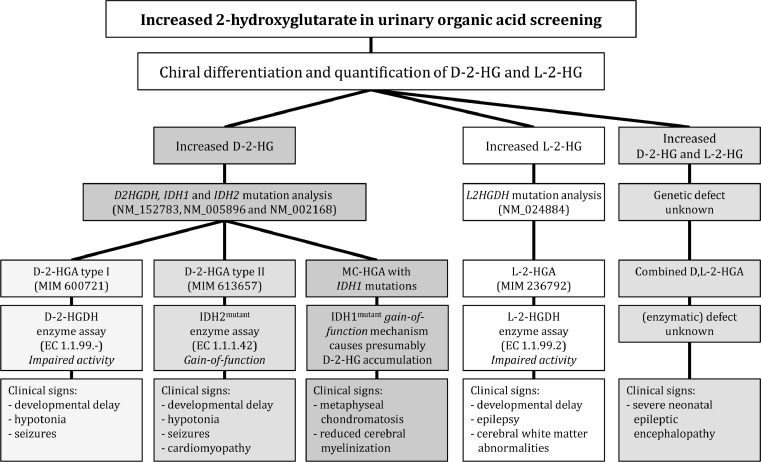
Diagnostic flow chart for 2-hydroxyglutaric acidurias

The inborn 2-hydroxyglutaric acidurias represent distinct neurometabolic disorders for which the underlying etiology is only beginning to be revealed (summarized in Table 10 and supplemented with a diagnostic flow chart in Fig. 8). Although the D-, L- and combined disorders appear to form distinct clinical diseases, we remain hampered in our understanding of their pathophysiology, because the role of the metabolites themselves remains unclear. Specific metabolic pathways for these chiral intermediates are largely unknown, and their role in the central nervous system remains to be defined. The development of models (yeast, zebrafish, mouse) in which the specific genes are ablated/mutated (*D2HGDH, L2HGDH, IDH1, IDH2*) may help us to understand the associated biochemistry. Nonetheless, the current work has set the foundation for a more comprehensive understanding of these disorders, based upon clinical, metabolic, enzymatic and genetic characterization. As is the case with most “new” neurometabolic disorders, the heterogeneity of phenotypic/genotypic presentations is likely to expand as our knowledge of the disorders grows. Accurate working models combined with low-threshold screening can provide clues to therapeutic strategies.

**Table 10:** Overview of 2-hydroxyglutaric acidurias

	D-2-HGA type I (MIM# 600721)	D-2-HGA type II (MIM# 613657)	L-2-HGA (MIM# 236792)	D,L-2-HGA
Metabolites mean (n); range *(controls)*	Increased D-2-HG	Increased D-2-HG	Increased L-2-HG	Increased D-2-HG and L-2-HG
Urine (mmol/mol creat.)				
D-2-HG *(6 (18); 2.8-17)*	969 (20); 103-2414	2486 (19); 448-11305	normal	496 (6); 228-750
L-2-HG *(6 (18); 1.3-19)*	normal	normal	1364 (27); 226-4299	161 (6); 64-247
Plasma (μmol/L)				
D-2-HG *(0.7 (10); 0.3-0.9)*	68 (7); 26-123	366 (9); 99-757	normal	2.6 (4); 2.2-3.2
L-2-HG *(0.6 (10); 0.5-1.0)*	normal	normal	39 (18); 7-84	2.0 (4); 1.1-3.0
CSF (μmol/L)				
D-2-HG *(0.1 (10); 0.07-0.3)*	13 (3); 6-18	79 (4); 30-172	normal	1.1 (3); 0.42-2.5
L-2-HG *(0.7 (10); 0.3-2.3)*	normal	normal	92 (12); 23-474	normal
Other metabolites	--	--	Lysine increased in plasma and CSF, mostly normal in urine	Inconsistently increased urinary 2-KG, succinate, fumarate and lactate
Enzyme	d-2-hydroxyglutarate dehydrogenase	Isocitrate dehydrogenase 2	l-2-hydroxyglutarate dehydrogenase	--
	D-2-HGDH	IDH2	L-2-HGDH	
	EC 1.1.99.-	EC 1.1.1.42	EC 1.1.99.2	
Defect mechanism	Impaired activity	Gain-of-function	Impaired activity	
Gene	*D2HGDH*	*IDH2*	*L2HGDH*	--
	GeneBank 728294	GeneBank 3418	GeneBank 79944	
	NM_152783	NM_002168	NM_024884	
	MIM# 609186	MIM# 147650	MIM# 609584	
Type of mutations	Heterogeneous	c.419G>A, R140Q	Heterogeneous	
		c.418C>G, R140G		
Trait	Autosomal recessive	Autosomal dominant	Autosomal recessive	
Clinical signs	Developmental delay	Developmental delay	Developmental delay	Severe neonatal epileptic encephalopathy
	Hypotonia	Hypotonia	Epilepsy	
	Seizures	Seizures	Cerebellar ataxia	
		Cardiomyopathy		
	Onset at 0-6 years	Onset at 0-2 years	Insidious onset in childhood	Onset in infancy
	Lifespan unknown	Shortened lifespan	Highly distinct brain MRI abnormalities	Shortened lifespan
	Brain MRI abnormalities	Brain MRI abnormalities		Brain MRI abnormalities
Therapeutic strategies	--	--	Riboflavin may improve symptoms	--
Cancer	--	Not reported, but	Increased incidence of brain tumors	--
		*IDH2* mutations in neoplastic disorders		
Key references	Achouri et al 2004	Kranendijk et al 2010b	Rzem et al 2004	Muntau et al 2000
	Wickenhagen et al 2009 Struys et al 2006 Kranendijk et al 2010a	Kranendijk et al 2011	Aghili et al 2009	Read et al 2005
			Kranendijk et al 2010a	
			Steenweg et al 2009; 2010

## Abbreviations

2-HG: 2-hydroxyglutaric acid (undifferentiated for chiral d- or l-form)
2-HGA: 2-hydroxyglutaric aciduria
D-2-HG: d-2-hydroxyglutaric acid
D-2-HGA: d-2-hydroxyglutaric aciduria
D-2-HGDH: d-2-hydroxyglutarate dehydrogenase enzyme
*D2HGDH*: gene encoding d-2-hydroxyglutarate dehydrogenase
L-2-HG: l-2-hydroxyglutaric acid
L-2-HGA: l-2-hydroxyglutaric aciduria
L-2-HGDH: l-2-hydroxyglutarate dehydrogenase enzyme
*L2HGDH*: gene encoding l-2-hydroxyglutarate dehydrogenase
D,L-2-HGA: combined d,l-2-hydroxyglutaric aciduria
*IDH2*: gene encoding isocitrate dehydrogenase 2
2-KG: 2-ketoglutaric acid
HOT: hydroxyacid-oxoacid transhydrogenase enzyme
L-malDH: l-malate dehydrogenase enzyme
MIM: Mendelian Inheritance in Man
CSF: cerebrospinal fluid
